# Decreased TCF1 and BCL11B expression predicts poor prognosis for patients with chronic lymphocytic leukemia

**DOI:** 10.3389/fimmu.2022.985280

**Published:** 2022-09-23

**Authors:** Taotao Liang, Xiaojiao Wang, Yanyan Liu, Hao Ai, Qian Wang, Xianwei Wang, Xudong Wei, Yongping Song, Qingsong Yin

**Affiliations:** Department of Hematology, The Affiliated Cancer Hospital of Zhengzhou University and Henan Cancer Hospital, Zhengzhou, China

**Keywords:** chronic lymphocytic leukemia, T cell immune, TCF1, BCL11B, TTFT, prognosis

## Abstract

T cell immune dysfunction is a prominent characteristic of chronic lymphocytic leukemia (CLL) and the main cause of failure for immunotherapy and multi-drug resistance. There remains a lack of specific biomarkers for evaluating T cell immune status with outcome for CLL patients. T cell factor 1 (TCF1, encoded by the *TCF7* gene) can be used as a critical determinant of successful anti-tumor immunotherapy and a prognostic indicator in some solid tumors; however, the effects of TCF1 in CLL remain unclear. Here, we first analyzed the biological processes and functions of TCF1 and co-expressing genes using the GEO and STRING databases with the online tools Venny, Circos, and Database for Annotation, Visualization, and Integrated Discovery (DAVID). Then the expression and prognostic values of TCF1 and its partner gene B cell leukemia/lymphoma 11B (BCL11B) were explored for 505 CLL patients from 6 datasets and validated with 50 CLL patients from Henan cancer hospital (HNCH). TCF1 was downregulated in CLL patients, particularly in CD8+ T cells, which was significantly correlated with poor time-to-first treatment (TTFT) and overall survival (OS) as well as short restricted mean survival time (RMST). Function and pathway enrichment analysis revealed that TCF1 was positively correlated with BCL11B, which is involved in regulating the activation and differentiation of T cells in CLL patients. Intriguingly, BCL11B was highly consistent with TCF1 in its decreased expression and prediction of poor prognosis. More importantly, the combination of TCF1 and BCL11B could more accurately assess prognosis than either alone. Additionally, decreased TCF1 and BCL11B expression serves as an independent risk factor for rapid disease progression, coinciding with high-risk indicators, including unmutated IGHV, TP53 alteration, and advanced disease. Altogether, this study demonstrates that decreased TCF1 and BCL11B expression is significantly correlated with poor prognosis, which may be due to decreased TCF1+CD8+ T cells, impairing the effector CD8+ T cell differentiation regulated by TCF1/BCL11B.

## Background

Chronic lymphocytic leukemia (CLL) is a heterogenous B cell malignancy that is the most common adult leukemia in Western countries (1, 2). CLL cell survival depends on the tumor microenvironment ifimmu.2022.985280fimmu.2022.985280n which B cell receptor (BCR) signaling is highly activated by crosstalk between CLL cells and microenvironment-supporting cells, particularly T cells (3–5). T cells are the main effector cells involved in anti-tumor immunity, and several studies have shown enrichment of CD4+ and CD8+ T cells in CLL (6). Nevertheless, CD4+ T cells could stimulate CLL cell survival and proliferation *via* cytokine secretion and direct contact (5, 6), and CD8+ T cells are persistently stimulated in the CLL microenvironment and gradually become exhausted, finally losing effector function, particularly during disease progression (7). Exhaustion has been suggested to be causative of the poor response to chimeric antigen receptor T cell (CAR-T) therapies for CLL patients (5, 8, 9). However, little is known about specific indicators for evaluating the T cell immune status and its correlation with the prognosis of patients with CLL.

T cell factor-1 (TCF1) is a transcription factor encoded by the transcription factor 7 (*TCF7*) gene (10). As one of the critical master regulators for T cell commitment in the thymus, TCF1 is the first T cell-specific transcription factor induced and activated by Notch signaling in the successive stage of T lineage specification, and it maintains a high expression level until T cell maturation (11, 12). Activated TCF1 positively regulates two major target genes, B cell leukemia/lymphoma 11B (BCL11B) and GATA binding protein 3 (GATA3), to sustain T cell commitment and proliferation (13–17). Additionally, as pivotal T cell-specific transcription factors, TCF1 and BCL11B also participate in T cell activation and expansion (15, 18–20). More importantly, TCF1 and BCL11B are crucial for maintaining the stem-like properties of CD8+ T cells (21–24). Upon stimulation, TCF1 promotes CD8+ T cells to differentiate into TCF1+CD8+ T cells that participate in effective antitumor immunity (24, 25). Recently, infiltration of TCF1+CD8+ T cells into tumor tissues has been reported in several solid tumors (23, 24, 26). TCF1+ tumor-infiltrating lymphocytes (TILs) have been positively correlated with tumor regression, successful response to anti-PD-1 treatment, and longer overall survival (OS) (27, 28). Multiple studies have also found that decreased BCL11B expression predicts inferior clinical outcome in adult standard risk T cell acute lymphoblastic leukemia and myelodysplastic syndrome (29, 30). However, the effects of TCF1 and BCL11B expression on the prognosis of CLL patients remain unclear.

In this study, we first performed a variety of bioinformatic analysis of TCF1 expression and examined the effects of TCF1 on the time-to-first treatment (TTFT), overall survival (OS) and restricted mean survival time (RMST) for patients with CLL from public datasets. Furthermore, a protein-protein interaction (PPI) network of co-expressing genes was constructed using STRING, and we performed KEGG pathway and biological process analysis of significant, *TCF7*-related co-expressing genes using the Database for Annotation, Visualization, and Integrated Discovery (DAVID) database. Lastly, the above findings were validated with 50 CLL patients from our clinical center Henan Cancer Hospital (HNCH), revealing that both TCF1 and BCL11B participate in the regulation of T cell immunity and further determine the prognosis of patients with CLL.

## Methods

### Patients and clinical data collection

Peripheral blood samples from 50 patients with CLL, including 32 newly diagnosed patients and 18 refractory/relapsed (R/R) patients, and 8 age-matched healthy individuals (HIs) were analyzed after obtaining informed consent according to the hospital Medical Ethical Committee. Peripheral blood mononuclear cells (PBMCs) were isolated using Human Mononuclear Cell Separation Medium 1.077 (Bio-Processing System, 25610) density-gradient centrifugation, and they were cryopreserved until analysis. Clinical characteristics of 50 patients with CLL in the HNCH were listed in **Table S1**.

### Flow cytometry

Cells were washed in phosphate-buffered saline containing 2% FBS and incubated at 4°C for 30 min with combinations of the following antibodies: CD3-APC-A750 (A94680, Beckman Coulter), CD4-KrO (A96417, Beckman Coulter), CD8-APC-A700 (B49181, Beckman Coulter), and TCF1-PE (655208, Biolegend). Relevant isotype control mAbs were purchased from BD Biosciences. After two washes in phosphate-buffered saline containing 2% FBS, cells were analyzed by FACS Calibur (Becton Dickinson, San Jose, CA). Data were processed with Navios Flow Cytometer software (Beckman Coulter, Brea, CA, USA).

### GEO database analysis

The gene expression profiles of CLL cells and HIs were queried in the Gene Expression Omnibus (GEO) database, and the GSE19147 (31), GSE66425 (32), and GSE50006 datasets (33) were obtained. The GSE19147 dataset included 25 CLL patients and 8 HIs. GSE66425 included 30 PBMC samples from CLL patients and 5 samples of PBMCs from HIs, and GSE50006 included 32 B cell samples from HIs and 188 CLL samples. Differences in expression between CLL patients and HIs were compared using GEO2R.

### mRNA expression analysis

To verify the data from the public datasets, total RNA was prepared from CLL patients and HIs using TRIzol Reagent (Invitrogen, Carlsbad, CA, USA) according to the manufacturer’s instructions. cDNA was synthesized from equal amounts of total RNA (1 μg) using HiScript^®^ III RT SuperMix for real-time quantitative PCR (qPCR) (+gDNA wiper) (Vazyme, Q711-02, China) and analyzed by ChamQ Universal SYBR qPCR Master Mix. **Table S2** lists the primers used for PCR, which was performed using the ABI 7500 FAST real-time PCR system. The levels of transcripts were quantified by the 2^-ΔΔCT^ (cycle threshold) method.

### TTFT and OS analysis of CLL patients

The TTFT of CLL patients was queried in the GSE39671 dataset (34), which included 130 CLL patients and their TTFT information. In addition, we collected samples from 43 CLL patients from HNCH to confirm the TTFT results. The TTFT and OS for CLL patients is shown in Kaplan-Meier (KM) survival curves and analyzed by the log-rank Kaplan-Meier method using the “survival” package in R software (version 4.1.0). CLL prognoses were queried in the GSE22762 dataset (35), which included 107 CLL patients and OS information. CLL patients were segregated into 2 distinct categories according to TCF1 expression. The cutpoint value was calculated by the “survminer” package in R, and the RMST was obtained by the “survRM2” package in R. All cutpoint values are shown in **Figure S1**.

### Co−expressed gene calculation and analysis

The genes differentially expressed in correlation with *TCF7* in the GSE39671 and GSE22762 datasets were analyzed by the Spearman correlation coefficient. Additionally, the Venny 2.1.0 program was used to screen for common genes in the two databases (R > 0.3 and *P* < 0.05), and the DAVID version 6.8 (36) was used to conduct corresponding biological process and KEGG pathway enrichment analysis, and a bubble plot was drawn with the “ggplot2” package in R.

### PPI network construction and cluster identification

A PPI network for co-expressing genes was constructed using the Search Tool for the Retrieval of Interacting Genes (STRING) database (https://string-db.org/cgi/) (37), and the results were visualized using Cytoscape software (version 3.4.0) (38). The cutoff criterion for the confidence score was the default setting (> 0.7). In addition, the closely connected protein-interactive regions were obtained by the Molecular Complex Detection (MCODE) plug-in, and the correlation of hub genes in GSE39671 and GSE22762 was plotted with the Circos online tool, and the biological process results were also shown in a PPI network by the STRING database. Further, the hub genes from MCODE were filtered again using the Markov Clustering (MCL) algorithm.

### Statistical analysis

Data were analyzed using GraphPad Prism 8.2.1. Results are presented as the mean ± SD. Student’s unpaired t-test was used for differential expression analysis, the log-rank test was used to indicate the statistical significance of survival or TTFT correlation between groups, and the correlation of two genes was tested by the Spearman correlation coefficient. Survival curves were analyzed by the log-rank Kaplan-Meier method. Cox regression analysis was constructed to determine the hazard ratio (HR). All statistically significant variables (*P* < 0.05), as found in the univariate analyses, were included in multivariate analysis based on a Cox proportional hazards model. *P* < 0.05 was considered statistically significant.

## Results

### TCF1 expression significantly decreases in CLL patients

**Figure 1:**
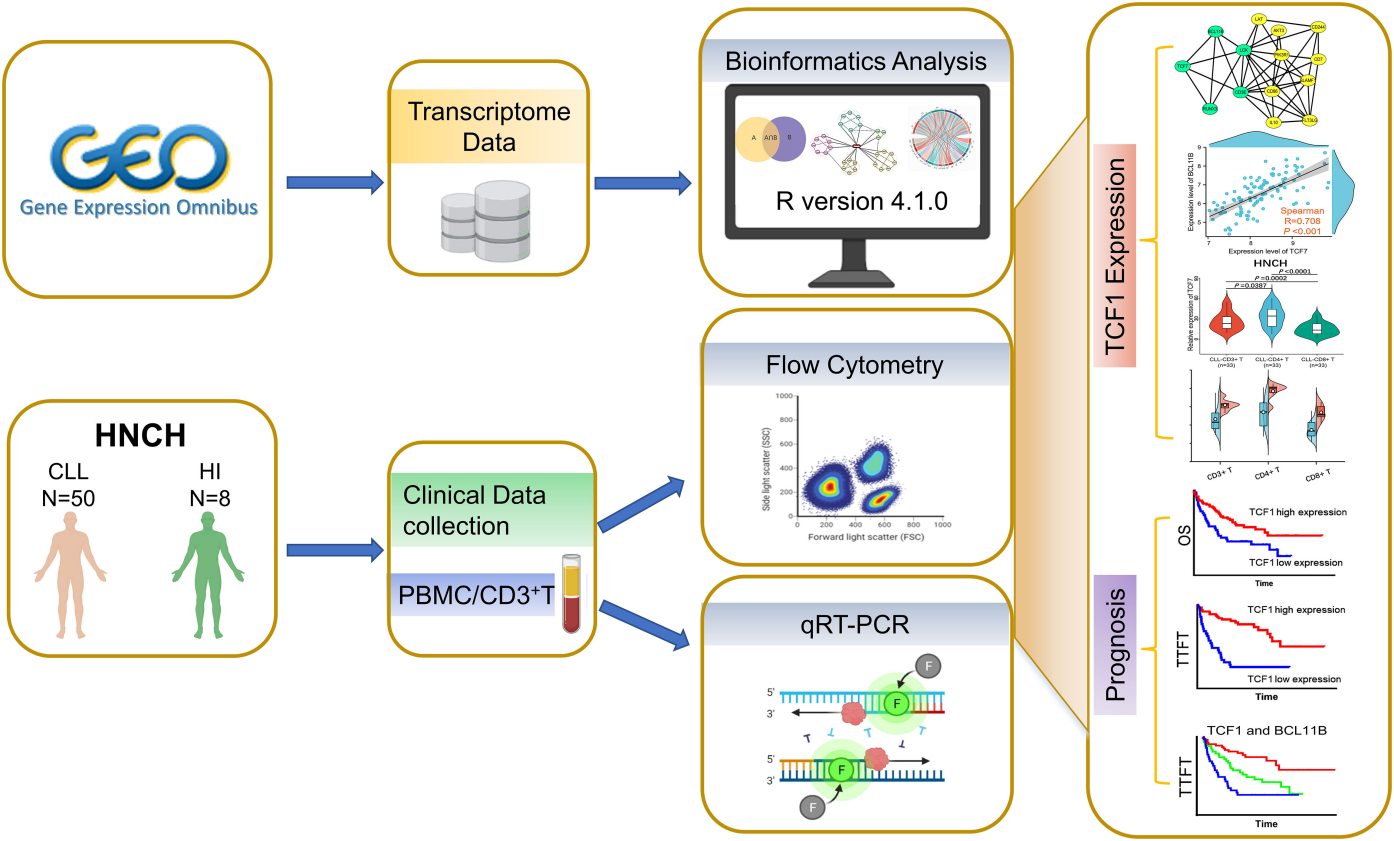
Study flow chart. The expression, biological function, and effects of TCF1 and its co-expressing genes were analyzed in the GEO and STRING databases. Then, the above findings were validated in 50 CLL patients from Henan Cancer Hospital (HNCH).

**Figure 2:**
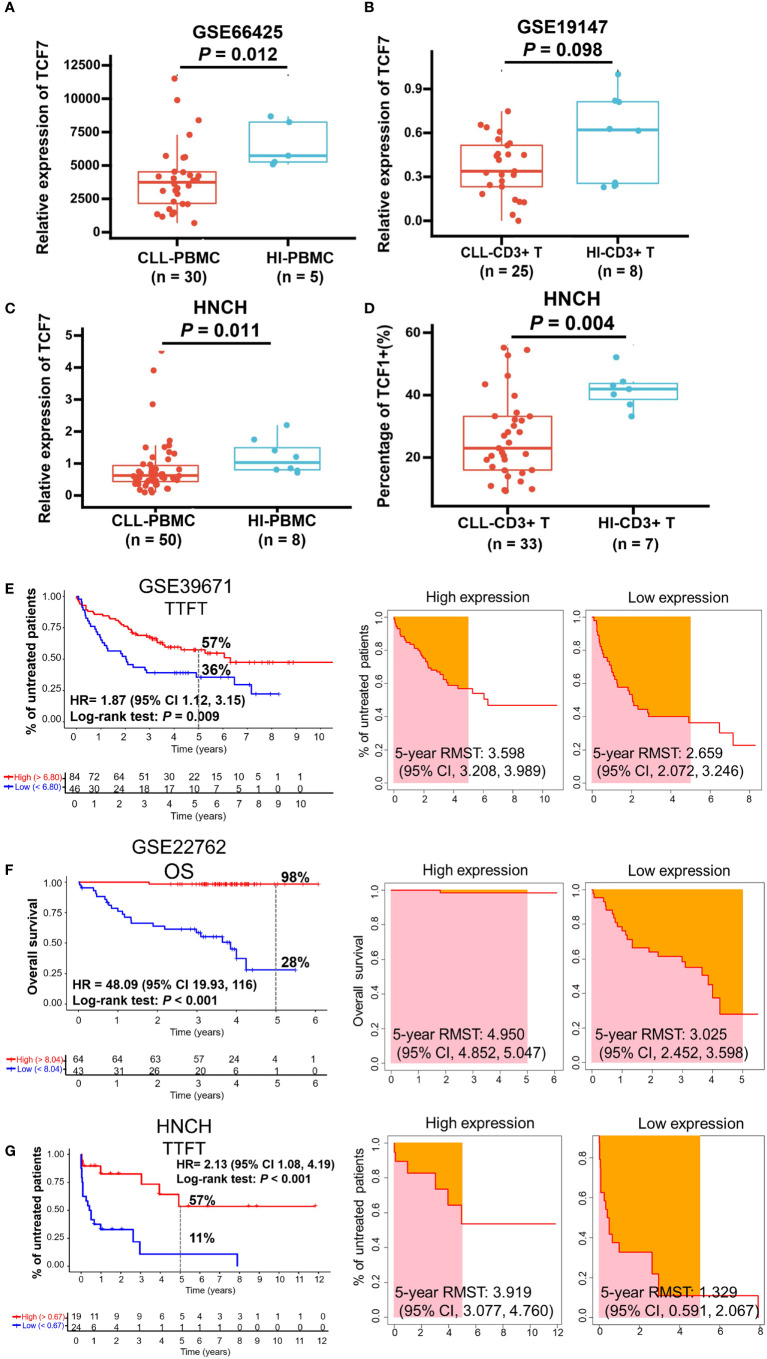
The expression and predictive effects of TCF1 on the TTFT and OS for patients with CLL. The expression of TCF1 in PBMCs and CD3+ T cells from CLL patients and healthy individuals (HIs) in the GSE66425 **(A)** and GSE19147 **(B)** datasets. **(C)** qPCR and **(D)** FCM verified the results of TCF1 expression in PBMCs and CD3+ T cells from CLL patients and HIs in the HNCH. **(E)** The TTFT (left panel) and RMST (right panel) for the high and low TCF1 expression groups in the GSE39671 dataset. **(F)** The OS (left panel) and RMST (right panel) for the high and low TCF1 expression groups in the GSE22762 dataset. **(G)** The TTFT (left panel) and RMST (right panel) for the high and low TCF1 expression groups in the HNCH. Error bars indicate SE.

To investigate the effects of TCF1 in CLL, we developed the research plan shown in the study flow chart (**Figure 1**). We first analyzed the expression of the TCF1, in PBMCs, CLL cells, T cells, and normal B cells in CLL patients from the GEO database and HNCH, respectively. The expression of TCF1 in PBMCs was mainly concentrated in T cells, it was quite low in CLL cells, and it was lower in normal B cells (**Figures S2A**, **S2B**). Moreover, TCF1 expression in PBMCs from CLL patients was lower than that in HIs in the GSE66425 dataset (*P* = 0.012; **Figure 2A**). Although there was no significant difference compared with HIs in the GSE19147 dataset, the proportion of TCF1+CD3+ T cells in CLL patients displayed a clear downward trend (*P* = 0.098; **Figure 2B**). We verified the above results in 50 patients with CLL from HNCH. Amazingly, TCF1 had low expression not only in PBMCs as determined by qPCR (*P* = 0.011; **Figure 2C**) but also in CD3+ T cells as determined by FCM (*P* = 0.004; **Figure 2D**) in CLL patients compared with HIs, which is highly consistent with the above findings from the GEO databases. Therefore, compared with HIs, TCF1 was significantly downregulated in the PBMCs of CLL patients, particularly in CD3+ T cells.

### Decreased TCF1 expression predicts short TTFT and OS for CLL patients

Most patients with CLL are diagnosed at the early stage of asymptomatic disease and are not treated until treatment indicators appear. TTFT is an important index that evaluates disease stability in patients with CLL like lymphocyte doubling time (39). Thus, we explored the predictive effects of TCF1 on the TTFT and OS of patients with CLL using the GSE39671 and GSE22762 datasets, respectively. The results demonstrated that CLL patients with low TCF1 expression have a shorter TTFT (5-year TTFT rate: 36% *vs.* 57%, *P* = 0.009; **Figure 2E**) and OS (5-year OS rate: 28% *vs.* 98%, *P* < 0.001; **Figure 2F**) than those with high TCF1 expression. Next, we confirmed the above findings in 43 CLL patients from HNCH and found that low TCF1 expression appeared to be correlated with short TTFT (5-year TTFT rate: 11% *vs.* 57%, *P* < 0.001; **Figure 2G**). Furthermore, we employed RMST to confirm the TTFT and OS data and found that patients with low TCF1 expression have a shorter RMST than those with high TCF1 expression (**Figures 2E–G**, right panel). Collectively, there was a clear trend where patients with low TCF1 expression have rapid disease progression and a short survival time. Thus, TCF1 can be used as a predictive biomarker of inferior prognosis for CLL patients.

### Construction of a *TCF7* co−expressing gene PPI network

**Figure 3:**
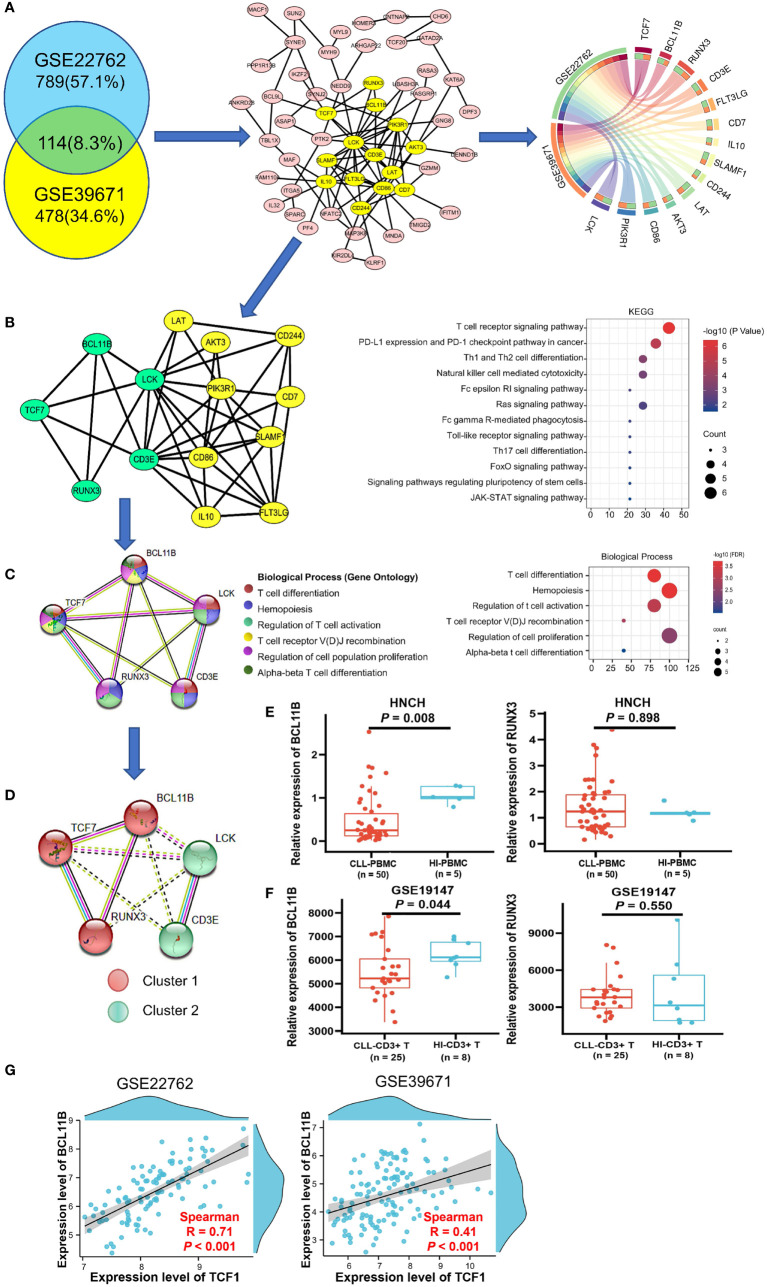
Biological process and KEGG pathway analysis of *TCF7* co-expressing genes and partner gene screening in patients with CLL. **(A)** The 114 common co-expressing genes (R > 0.3 P < 0.05) in the GSE22762 and GSE39671 datasets were filtered by the Venny online tool (left panel). Then, the STRING online tool was used to create the PPI network, MCODE analysis (middle panel) identified the 14 hub genes (highlighted in yellow), and the correlations of the 14 hub genes in the GSE2276 and GSE39671 datasets were plotted by the Circos web tool (right panel). **(B)** The PPI network of the 14 hub genes and their KEGG pathway annotation. **(C)** The PPI network and biological processes of 5 hub genes was directly related to *TCF7*. **(D)** MCL clustering analysis demonstrated a close relationship among *TCF7*, *BCL11B*, and *RUNX3* in CLL patients in the GSE22762 and GSE39671 datasets. The expression of *BCL11B* and *RUNX3* was measured in CLL patients from the HNCH **(E)** and GSE19147 **(F)** dataset, respectively. **(G)** The correlation between *TCF7* and *BCL11B* in the GSE39671 and GSE22762 datasets, respectively. R, Spearman correlation coefficient. Error bars indicate SE.

The 114 genes commonly co-expressed with *TCF7* in the GSE39671 (592 genes) and GSE22762 datasets (903 genes) (**Figure 3A**, left panel) were filtered by Venny and then built into a protein-protein network using the STRING database. Cytoscape (MCODE plug-in) was used to establish the most important module, which is highlighted in yellow (**Figure 3A**, middle panel). Based on the degree score, we selected the module with the highest score that included 14 genes: *TCF7*, *BCL11B*, *RUNX3*, *LCK*, *CD3E*, *LAT*, *AKT3*, *PIK3R1*, *CD86*, *IL10*, *FLT3LG*, *SLAMF1*, *CD7*, and *CD244*. These genes were identified as potential hub genes, and the expression levels of the 14 hub genes were plotted by the Circos webtool for the GSE22762 and GSE39671 datasets (**Figure 3A**, right panel).

### Functional and KEGG pathway analysis of the 14 hub genes in CLL

The results of KEGG pathway analysis, which was performed using the DAVID database and plotted by R, indicated that the 14 hub genes mainly participate in T cell receptor signaling and T cell differentiation (**Figure 3B**, right panel). Notably, we found that, of the 14 genes in the PPI network, *BCL11B*, *RUNX3*, *CD3E*, and *LCK* are directly related to *TCF7* gene (highlighted in green) (**Figure 3B**, left panel). We next reconstructed the PPI network of these five genes using the STRING database (**Figure 3C**, left panel). The biological process category of each gene is displayed in different colors and mainly include regulation of T cell activation, T cell differentiation, and T cell receptor recombination (**Figure 3C**, right panel), which is highly consistent with the above KEGG analysis results. These data indicate that *TCF7* coordinates with hub genes in regulating T cell immunity.

To determine genes closely related to *TCF7*, we clustered the 5 genes using the MCL method. Remarkably, the 5 genes divided into 2 clusters, cluster 1 (including *TCF7*, *RUNX3*, and *BCL11B*) and cluster 2 (including *LCK* and *CD3E*). It was evident that the genes in cluster 1 were closely related to *TCF7* (**Figure 3D**). We further verified the expression of *BCL11B* and *RUNX3* in PBMCs from CLL patients (HNCH) and HIs by qPCR. Intriguingly, *BCL11B* expression was significantly decreased in CLL patients compared with that in HIs (*P* = 0.008; **Figure 3E**, left panel), but there was no significant difference in *RUNX3* between CLL patients and HIs (*P* = 0.898, **Figure 3E**, right panel). Likewise, we found that *BCL11B* expression in CD3+ T cells was significantly decreased in CLL-CD3+ T cells compared to HI-CD3+ T cells in the GSE19147 dataset (*P* = 0.044 **Figure 3F**, left panel), but *RUNX3* did not demonstrate a difference between CLL and HIs (*P* = 0.550, **Figure 3F**, right panel). In addition, there was a high positive correlation between *TCF7* and *BCL11B* expression in the GSE22762 (R = 0.71, *P* < 0.001) and GSE39671 (R = 0.41 P < 0.001) datasets (**Figure 3G**). Therefore, among the genes co-expressed with *TCF7* in CLL patients, *BCL11B* may be the closest partner gene for *TCF7* involved in T cell immune regulation.

### Decreased BCL11B expression predicts short TTFT and OS for CLL patients

**Figure 4:**
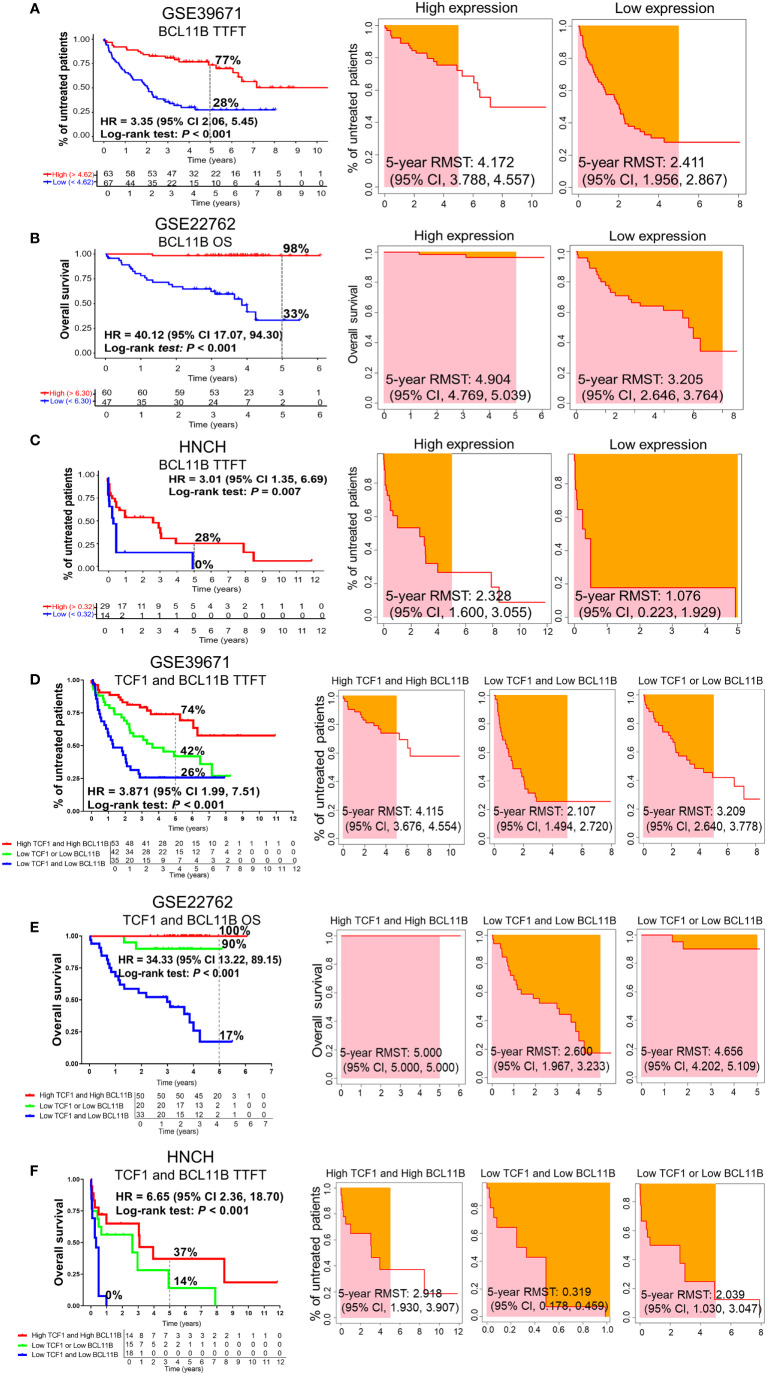
The predictive effects of BCL11B alone and in combination with TCF1 on the TTFT and OS of patients with CLL. **(A)** The TTFT (left panel) and RMST (right panel) for the high and low BCL11B expression groups in the GSE39671 dataset. **(B)** The OS (left panel) and RMST (right panel) for the high and low BCL11B expression groups in the GSE22762 dataset. **(C)** The TTFT (left panel) and RMST (right panel) for the high and low BCL11B expression groups in the HNCH. **(D)** The effects of TCF1 combined with BCL11B on TTFT (left panel) and RSMT (right panel) in the GSE39671 dataset. **(E)** The effects of TCF1 combined with BCL11B on OS (left panel) and RSMT (right panel) in the GSE22762 dataset. **(F)** The effects of TCF1 combined with BCL11B on TTFT (left panel) and RSMT (right panel) in the HNCH.

To investigate the effects of BCL11B alone or in combination with TCF1 on the prognosis of patients with CLL, we further analyzed the effects of BCL11B on TTFT and OS for CLL patients in the GSE39671 and GSE22762 datasets, respectively (**Figures S3A**, **S3B**). Low expression of BCL11B was significantly associated with shorter TTFT and poorer OS compared with high expression (5-year TTFT: 28% *vs.* 77%; 5-year OS: 33% *vs.* 98%) (**Figures 4A, B**, left panel). Similarly, patients with low BCL11B expression had shorter RMST than those with high expression (**Figures 4A, B**, right panel). As expected, the above findings were confirmed with 50 patients with CLL from HNCH (5-year TTFT: 0% *vs.* 28%) (**Figure 4C**). Next, we analyzed the effects of BCL11B in combination with TCF1 on prognosis, compared with those who were TCF1^high^BCL11B^high^, CLL patients who were TCF1^low^BCL11B^low^ had a poorer TTFT and OS (**Figures 4D, E**, left panel) as well as shorter RMST (**Figures 4D, E**, right panel). Specifically, the 5-year TTFT was 26% and 74%, and the 5-year OS was ≤ 17% and 100%, respectively. Similarly, those results also were validated in HNCH, the 5-year TTFT and for TCF1^low^BCL11B^low^ and TCF1^high^BCL11B^high^ was 0% and 37%, respectively (**Figure 4F**). Therefore, BCL11B can be used as a predictive biomarker for inferior prognosis for CLL patients. More importantly, the combination of TCF1 and BCL11B expression could more accurately assess the prognosis of CLL patients compared with either alone.

### Correlation between TCF1 expression and clinical factors for CLL patients

**Figure 5:**
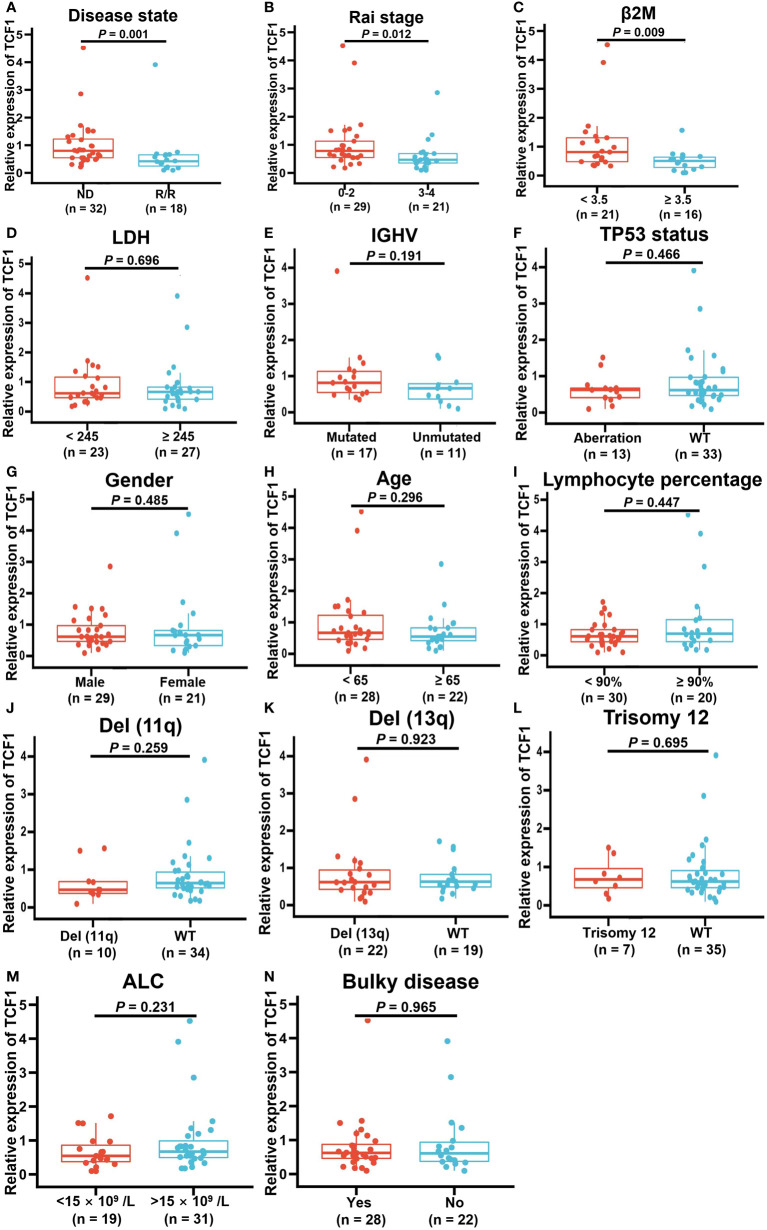
Correlation between decreased TCF1 expression and clinical factors in CLL patients. The correlation of TCF1 expression and various clinical characteristics, such as **(A)** newly diagnosed (ND) and relapsed/refractory (R/R) patients, **(B)** Rai stage 0-2 and 3-4, **(C)** β2M < 3.5 or ≥ 3.5 (mg/L), **(D)** LDH < 245 or ≥ 245 (IU/L), **(E)** IGHV mutated and unmutated, **(F)** TP53 aberration or WT, **(G)** Male or female CLL patients, **(H)** < 65 or ≥ 65 years old, **(I)** Lymphocyte percentage, **(J)** Del(11q) or WT, **(K)** Del(13q) or WT, **(L)** Trisomy12 or WT, **(M)** ALC < 15 ×10^9^/L or >15 ×10^9^/L, and **(N)** Bulky or no. WT means wild type. Del means deletion. Error bars indicate SE.

To explore the correlation between TCF1 expression and the characteristics of patients with CLL, we next integrated a series of clinical characteristics, including disease state, Rai stage, β2 microglobulin (β2M) level, lactate dehydrogenase (LDH) level, gender, age, lymphocyte percentage, IGHV mutation status, cytogenetic abnormalities such as del(17p) or P53 mutation (TP53 aberration), del(11q), del(13q) and trisomy12, absolute lymphocyte count (ALC), and bulky disease (≥ 5 cm) (**Figures 5A–N**). Notably, decreased TCF1 expression was significantly associated with relapsed and refractory disease (*P* = 0.001; **Figure 5A**), Rai stage 3-4 (*P* = 0.012; **Figure 5B**), and β2M ≥ 3.5 (mg/L) (*P* = 0.009; **Figure 5C**), which mainly serve as clinical indexes for disease progression and adverse prognosis. In relapsed and refractory CLL patients, we found there was no significant difference in the effect of first-line treatment regimens on TCF1 expression except for a slight upward trend in the ibrutinib group, which may be related to the small number of cases in different groups (**Table S1**).

Cox regression analysis demonstrated that low TCF1 and BCL11B expression are independent predictive factors for short TTFT of CLL patients (**Table 1**). Specifically, in univariate analysis, ≥ 65 years old, female, high β2M level, high LDH level, unmutated IGHV, TP53 aberration, del(11q), trisomy12, Rai stage 3-4, R/R, low TCF1, and low BCL11B were significant risk factors for short TTFT. Statistically significant factors for TTFT (*P* < 0.05) were included in the multivariate analysis, revealing that unmutated IGHV, TP53 aberration, trisomy12, Rai stage 3-4, R/R, low TCF1, and low BCL11B were independent risk factors for shortened TTFT. Thus, TCF1 is a potential clinical biomarker for predicting disease progression for CLL patients.

**Table 1:** Univariate and multivariate Cox regression analysis of risk factors associated with TTFT.

VARIABLES	UNIVARIATE COX		MULTIVARIATE COX	
	HR (95% CI)	*P* value	HR (95% CI)	*P* value
Age (< 65 vs. ≥ 65)	0.37 (0.17 - 0.83)	0.001	0.90 (0.72 - 1.14)	0.385
Gender (Male vs. Female)	0.24 (0.10 - 0.58)	0.001	1.47 (0.09 - 23.08)	0.785
β2M (< 3.5 vs. ≥ 3.5)	0.36 (0.15 - 0.85)	0.002	0.01 (0.00 - 1.02)	0.051
LDH (< 245 vs. ≥ 245)	0.49 (0.22 - 1.07)	0.041	0.07 (0.00 - 8.10)	0.268
IGHV (Unmut vs. Mut)	3.29 (1.03 - 10.56)	0.004	0.00 (0.00 - 0.28)	0.020
TP53 aberration (No vs. Yes)	0.47 (0.20 - 1.12)	0.030	0.00 (0.00 - 0.14)	0.008
Lymphocyte Percentage (< 90% vs. ≥ 90%)	1.14 (0.58 - 2.23)	0.703		
Del(13q) (No vs. Yes)	1.63 (0.74 - 3.59)	0.178		
Del(11q) (No vs. Yes)	0.48 (0.18 - 1.24)	0.040	1.57 (0.01 - > 50)	0.877
Trisomy 12 (No vs. Yes)	0.35 (0.11 - 1.08)	0.006	45.99 (1.69 - > 50)	0.023
Rai stage (0-2 vs. 3-4)	0.36 (0.17 - 0.75)	< 0.001	30.81 (2.59 - > 50)	0.007
Disease status (ND vs. R/R)	0.37 (0.17 - 0.83)	0.001	> 50 (5.85 - > 50)	0.007
ALC (< 15 × 10^9^/L *vs.* > 15 ×10^9^/L)	0.61 (0.31 - 1.20)	0.141		
Bulky Disease (Yes or No)	1.12 (0.57 - 2.19)	0.737		
TCF1 (Low vs. High)	4.18 (1.8 - 9.85)	< 0.001	0.00 (0.00 - 0.13)	0.006
BCL11B (Low vs. High)	2.37 (1.06 - 5.27)	0.007	> 50 (1.79 - > 50)	0.028

CI, confidence interval; HR, hazard ratio.

### Percentage of TCF1+ cells in the CD3+, CD4+, and CD8+ T cell subgroups from CLL patients

**Figure 6:**
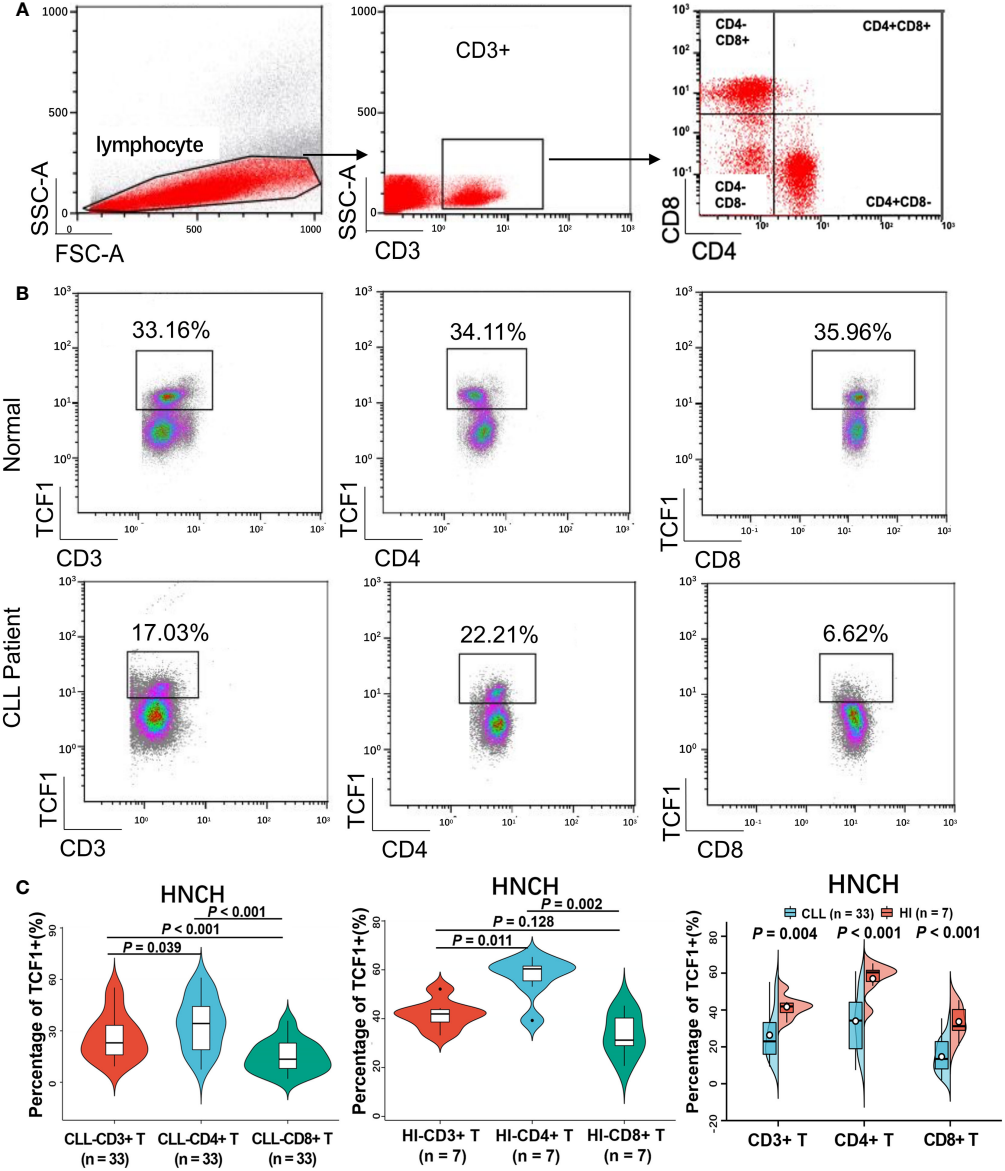
The percentage of TCF1+ cells on various T cell subgroups of CLL and HIs. **(A)** Flow cytometry gating strategy. **(B)** Representative profiles of TCF1 expression on CD3+, CD3+CD4+, and CD3+CD8+ T cells in HIs (top) and CLL patients (bottom). **(C)** The expression of TCF1 in CD3+, CD3+CD4+, and CD3+CD8+ T cells from CLL patients and HIs in the HNCH. Error bars indicate SE.

To explore the reduced expression of TCF1 in various T cell subgroups, we subsequently detected the percentage of TCF1+ cells in the CD3+, CD3+CD4+, and CD3+CD8+ T cell subgroups of 33 CLL patients and compared these with HIs. We restricted all analyses to CD3+ T cells (**Figure 6A**). The results demonstrated that the percentages of TCF1+ cells in the CD3+, CD3+CD4+, and CD3+CD8+ T cell populations from CLL patients were significantly lower than that in corresponding T cell subgroups from HIs (*P* values are 0.004, < 0.001 and < 0.001, respectively), particularly for CD3+CD8+ T cells (**Figures 6B, C**). We further compared the percentages of TCF1+ cells among the different T subgroups and found that TCF1+ cells had a higher percentage in CD3+CD4+ T cells irrespective of CLL patients or HIs (**Figure 6C**). Notably, the percentage of TCF1+ cells in the CD3+CD8+ T cell population was significantly lower than that in the CD3+CD4+ T cell population, particularly in CLL patients. It has been reported that TCF1+CD8+ T cells promote effective antitumor immunity (40). Based on previous studies and our current findings, decreased TCF1+CD8+ T cells in CLL patients indicates T cell immune dysfunction. Therefore, TCF1 has the potential to be a biomarker for T cell immune status and a therapeutic target in CLL patients.

## Discussion

Accumulating studies have illustrated that TCF1 is a key regulator for maintaining the stem-like properties of central memory CD8+ T cells and the cytotoxicity of effector CD8+ T cells (22, 41). TCF1+CD8+ T cells reportedly serve as a positive biomarker for prolonged survival and effective response to anti-PD-1 treatment in solid tumors (24, 27). However, little is known about the role of TCF1 in assessing the immune function with clinical outcome of CLL patients. In this study, TCF1 and BCL11B downregulation is a discernable event where decreased expression of these genes in CLL patients was significantly correlated with short TTFT and poor OS, particularly with the two combined. This lower expression may be due to the decrease in TCF1+CD8+ T cells, which thus impairs the differentiation of effector CD8+ T cells regulated by TCF1 and BCL11B.

In recent years, research on the role of TCF1 in evaluating efficacy and prognosis has exhibited an upward trend (21–24, 27, 41). Previous studies have claimed that infiltration of TCF1+CD8+ T cells contributes to the induction of tumor regression but not that of TCF1+CD4+ T cells (23). Herein, our findings demonstrate that the proportion of TCF1+ T cells, particularly in CD8+ T cells, is significantly decreased in CLL patients. As previously reported, TCF1 was highly expressed in naïve T cells and downregulated during effector differentiation, and low TCF1 expression was a characteristic of terminally differentiated T cells (42, 43). A recent study has also demonstrated that the terminally exhausted T cells generated in response to chronic viral infection lacked TCF1 expression and were TCF1 negative (41). Therefore, in this study, the decrease in TCF1 expression and proportion of TCF1+CD8+ T cells indicates the accumulation of terminally differentiated and exhausted T cells and T cell immunodeficiency, which is consistent with clinical findings for CLL patients (44), which in turn drives the CLL progression (3). In addition, it was reported that TCF1+CD8+ T cells contribute to broadening the diversity of the TCR repertoire (45). These decreased TCF1+CD8+ T cells suggest skewed T cell compartments and disordered T cell immunity, which explains the deficient anti-tumor effects of CD8+ T cells in CLL patients. Therefore, it is not surprising that low TCF1 expression indicates rapid progression and inferior prognosis for CLL patients.

To further explore the mechanism by which TCF1 regulates CLL prognosis and the partner molecules interacting with TCF1, functional and pathway enrichment analysis revealed that TCF1 is positively correlated with BCL11B, which is mainly involved in T cell differentiation and activation and immune regulation in CLL patients, in accordance with the literature (20, 22). Moreover, BCL11B was highly consistent with TCF1 in terms of decreased expression and the prediction of poor prognosis based on analysis of public datasets and HNCH data. Importantly, combination of the two genes could more accurately predict disease progression and prognosis for CLL patients compared with either alone. Cox regression analysis also demonstrated that both TCF1 and BCL11B downregulation could serve as an independent risk factor for rapid disease progression. Therefore, BCL11B may be a close partner for TCF1, and both may be used as indicators of T cell immunity to regulate the prognosis of CLL patients.

We additionally explored evidence that BCL11B cooperates with TCF1 to participate in immune regulation. Previous studies have reported that BCL11B plays an important role in mature T cell activation and proliferation (18, 21). In addition, BCL11B is a downstream target of TCF1 (15, 16), and TCF1 deficiency results in BCL11B downregulation (15, 16, 46). Moreover, another study demonstrated that BCL11B deficiency in virus-specific CD8+ T cells results in decreased memory precursor effector cells, and the effector cells skewed toward short-lived effector cells, resulting in a reduced ability to secrete cytotoxic granules (21). These data suggest an impairment in effector CD8+ T cell differentiation. These findings indicated that BCL11B is highly correlated with TCF1 and coordinates with this gene to maintain the stem-like properties and cytotoxicity of effector CD8+ T cells (22, 24, 41). Unfortunately, we found that TCF1 and BCL11B expression was decreased in CLL patients, and this resulted in impaired differentiation potential for effector CD8+ T cells (21, 22), which clinically manifested as the accumulation of terminally differentiated T cells (44), immune function dysfunction, and further rapid CLL progression. Although the data were not yet significant enough, this, to some extent, explains why decreased TCF1 and BCL11B expression leads to poor prognosis for patients with CLL. TCF1 and BCL11B may be promising biomarkers for T cell immune status and therapeutic targets in CLL patients.

Additionally, it is worth noting that, as an independent risk factor for rapid disease progression of CLL patients, decreased TCF1 and BCL11B expression coincided with high-risk indicators for CLL, including unmutated IGHV and TP53 aberration. Moreover, decreased TCF1 expression was significantly correlated with clinical indicators for CLL disease advancement (1), such as relapsed and refractory disease, Rai stage 3-4, and high β2M level. It has been reported that with the progression of CLL disease, T cell immune dysfunction is aggravated, which further leads to poor efficacy and treatment resistance (8, 9). Collectively, reduced TCF1 and BCL11B expression can be used as a clinical indicator for T cell immune function to assess the disease progression and prognosis of patients with CLL in the clinic.

## Conclusions

This study demonstrated for the first time that reduced TCF1 and BCL11B expression is significantly correlated with the poor prognosis of patients with CLL. TCF1 co-expression analysis revealed that TCF1 is positively associated with BCL11B, which is mainly involved in regulating the activation and differentiation of T cells. Downregulation of TCF1 and BCL11B, particularly in CD8+ T cells, in CLL patients may impair the stem-like differentiation potential of memory CD8+ T cells, leading to T cell immune dysfunction, and this may provide insight into the mechanism by which TCF1 and BCL11B regulates the prognosis of CLL patients. Therefore, manipulating the effects of TCF1 and BCL11B on T cell immunity will contribute to further designing combined therapies for T cell-based immunotherapy in the future.

## Data availability statement

Publicly available datasets were analyzed in this study. This data can be found here: https://www.ncbi.nlm.nih.gov/geo/. The original contributions presented in the study are included in the article/**Supplementary Material**. Further inquiries can be directed to the corresponding author.

## Ethics statement

The studies involving human participants were reviewed and approved by The ethics committees of the Affiliated Cancer Hospital of Zhengzhou University. The patients/participants provided their written informed consent to participate in this study.

## Author contributions

TTL analyzed the data, designed the figures, performed statistical analysis, and wrote the manuscript. XJW and YYL did flow cytometry and qPCR analysis. HA and QW collected the patient information and literature information. XWW carried out bioinformatics analysis. XDW and YPS designed and participated in editing the manuscript. QSY designed the study, analyzed the data, and wrote and revised the manuscript. All authors read and approved the final manuscript.

## Funding

This study was supported by the Natural Science Foundation of Henan province (162300410280), the Foundation for Young Teachers’ Basal Research of Zhengzhou University (jc202050015), and the Medical Science and Technology Research Project of Henan Province (LHGJ20220185).

## Acknowledgments

We are very thankful to Prof. Yangqiu Li (Jinan University, Guangzhou) for outstanding manuscript edits and her advice on bioinformatics analyses.

## Conflict of interest

The authors declare that the research was conducted in the absence of any commercial or financial relationships that could be construed as a potential conflict of interest.

## Publisher’s note

All claims expressed in this article are solely those of the authors and do not necessarily represent those of their affiliated organizations, or those of the publisher, the editors and the reviewers. Any product that may be evaluated in this article, or claim that may be made by its manufacturer, is not guaranteed or endorsed by the publisher.
