# Supplementary material for: Decreased TCF1 and BCL11B expression predicts poor prognosis for patients with chronic lymphocytic leukemia
Source: Front Immunol. 2022 Sep 23;13:985280. doi: 10.3389/fimmu.2022.985280 (PMC9539190; doi:10.3389/fimmu.2022.985280)
Supplement: Supplementary file 1 [file DataSheet_1.docx]

**Supplementary Information**

**“Decreased TCF1 and BCL11B expression predicts poor prognosis for patients with chronic lymphocytic leukemia”**

**Taotao Liang, Xiaojiao Wang, Yanyan Liu, et al:**

**Supplemental TABLE1** Clinical characteristics of 50 patients with CLL in the HNCH.

| **CHARACTERISTIC** | **N (FREQUENCY, %) OR MEDIAN (RANGE)** |
| --- | --- |
| Sex |  |
| Female | 21 (42.0) |
| Male | 29 (58.0) |
| Age (years) | 63 (35 - 91) |
| Rai stage |  |
| 0 | 2 (4.0) |
| I | 10 (20.0) |
| II | 17 (34.0) |
| III | 4 (8.0) |
| IV | 17 (34.0) |
| Cytogenetic abnormalities |  |
| TP53 aberration | 13 (26.0) |
| Del 11q | 10 (20.0) |
| Trisomy 12 | 7 (14.0) |
| Diploid | 1 (2.0) |
| Del 13q | 22 (44.0) |
| Survival status |  |
| Alive | 49 (98.0) |
| Dead | 1 (2.0) |
| IGHV |  |
| Mutated | 17 (34.0) |
| Unmutated | 11 (22.0) |
| Unknown | 22 (44.0) |
| β2M (mg/L) |  |
| ≥ 3.5 | 16 (32.0) |
| < 3.5 | 21 (42.0) |
| Unknown | 13 (26.0) |
| LDH (IU/L) |  |
| ≥ 245 | 27 (54.0) |
| <245 | 23 (46.0) |
| Disease status |  |
| Newly diagnosed | 32 (64.0) |
| Refractory/relapsed | 18 (36.0) |
| First-line therapy of Refractory/relapsed patients |  |
| Chemotherapy | 13 (72.2) |
| Ibrutinib | 2 (11.1) |
| Interferon | 1 (5.5) |
| Interferon combined with thalidomide | 1 (5.5) |
| Rituximab | 1 (5.5) |

**Supplemental TABLE 2** Real-time qPCR primers.

| **PRIMER NAME** | **PRIMER SEQUENCE (5’-3’)** |
| --- | --- |
| *TCF7* F | TCCACCACAGGAGGAAAAAGA |
| *TCF7* R | CGCAGGGCTAGTAAGCAGTT |
| *BCL11B* F | ATGTCCCGCCGCAAACAGG |
| *BCL11B* R | GGCTCGGACACTTTCCTGAGC |
| *RUNX3* F | TCAACGACCTTCGCTTCGTG |
| *RUNX3* R | ACCTTGATGGCTCGGTGGTA |
| *GAPDH* F | GAAGGTGAAGGTCGGAGTCAA |
| *GAPDH* R | GACAAGCTTCCCGTTCTCAG |

Abbreviations used: F, forward primer; R, reverse primer.

**Supplemental FIGURE 1** Optimal cutpoint value determination for the TCF7 and BCL11B genes. The optimal cutpoint values for TCF7 in the GSE39671 (A) and GSE22762 (B) datasets, and HNCH (C). And the cutpoint value of BCL11B in the GSE39671 (D) and GSE22762 (E) datasets, and HNCH (F). All the cutpoint value were determined by the “survminer” package in R.

**Supplemental FIGURE 2** The expression of TCF1 in CLL cells, T cells, and B cells from CLL patients. (A) The expression of TCF1 in T cells and CLL cells in CLL patients in the HNCH. (B) The expression of TCF1 in normal B cells and CLL cells in the GSE50006 dataset. Error bars indicate SE.

**Supplemental FIGURE 3** The effects of TCF1 and BCL11B alone or in combination on the TTFT and OS of CLL patients. (A) The predictive effects of TCF1 and BCL11B alone or in combination on the TTFT in the GSE39671 dataset. (B) The predictive effects of TCF1 and BCL11B alone or in combination on OS in the GSE22762 dataset.
